# mTOR Inhibition Rejuvenates the Aging Gingival Fibroblasts through Alleviating Oxidative Stress

**DOI:** 10.1155/2017/6292630

**Published:** 2017-07-19

**Authors:** Yiru Xia, Mengjun Sun, Yufeng Xie, Rong Shu

**Affiliations:** Department of Periodontology, School of Stomatology, Ninth People's Hospital, Shanghai Jiao Tong University School of Medicine, No. 639 Zhizaoju Road, Shanghai 200011, China

## Abstract

The aging periodontium may be vulnerable to periodontal pathogens and poor response to inflammation and susceptible to tumorigenesis. Human gingival fibroblasts (hGFs) through continuously replicative culture served as an in vitro surrogate for aging. To investigate the effects of the mechanistic target of rapamycin (mTOR) inhibition on the aging gingiva, we stimulated the high-passage hGFs with rapamycin (20 nmol/L) for 3 days and 30 days. The cellular and biological changes were examined by immunofluorescence, real-time PCR, ELISA, Western blotting, and flow cytometry. The data demonstrated that the inhibition of mTOR signaling led to fewer senescence-associated beta-galactosidase- (SA-*β*-Gal-) positive cells, delayed the onset of senescence, preserved the capability of proliferation, and lowered the expression levels of relevant senescence-associated markers, such as p16^INK4a^, p21^CIP1a^, interleukin-6 (IL-6), and IL-8. In addition, when infected by prominent periodontal pathogens, *Porphyromonas gingivalis* (ATCC 33277), rapamycin-pretreated groups decreased the expression of inflammatory cytokines (*IL-6* and *IL-8*) compared with the control group. mTOR inhibition upregulated the gene expression of antioxidant components (*Cat*, *Sod2*, and *Prdx3*; *P* < 0.05) and consequently neutralized the excessive reactive oxygen species (ROS). In conclusion, our results indicated that mTOR inhibition might rejuvenate the aging gingiva to some extent and relieve inflammation through eliminating oxidative stress.

## 1. Introduction

Aging, a significant topic of urgent worldwide concern, is related to the accumulated prevalence and severity of inflammatory and degenerative pathologies [1, 2]. The elderly also seem to have increased susceptibility to periodontitis [3–6]. The aging periodontal tissue (especially the aging gingiva) may react violently to periodontal pathogens (such as *Porphyromonas gingivalis* (*P. gingivalis*)). As the frontline of oral natural barriers, the aging gingiva may be vulnerable to periodontal diseases, poor response to inflammation, delay in wound healing, and tumorigenesis [7, 8]. Therefore, it is significant to find a useful method to delay or reverse the gingiva aging process.

Oxidative stress is strongly associated with aging and age-related diseases. The intracellular reactive oxygen species (ROS) accumulates during cellular senescence aggravating the destruction of the periodontium [9]. *P. gingivalis* infection might increase the intracellular ROS levels in human gingival epithelial cells [10].

The mechanistic target of rapamycin (mTOR) is a highly conserved serine/threonine kinase that serves a central role in integrating signals from regulating cell metabolism to various kinds of stress in eukaryotes [11]. Numerous studies demonstrated that mTOR inhibition seems to increase lifespan and delay the occurrence of aging-related diseases [12–14]. The effects of mTOR inhibition on aging periodontal tissue, however, remain to be explored.

Replicative senescence was used as an in vitro surrogate for aging [15]. With each replication, telomeres are shortened and cells eventually enter a stage of an irreversible proliferation arrest, termed replicative senescence [16, 17]. The aim of present study is to evaluate the effects of mTOR inhibition on preserving the proliferative potential, enhancing anti-inflammatory reaction and alleviating oxidative stresses on human gingival fibroblasts (hGFs). The results may provide a novel insight for periodontal disease treatment and a help for a further study on periodontal regeneration.

## 2. Materials and Methods

### 2.1. Cell Culture and mTOR Inhibition

Fresh tissue specimens (from 5 individuals aged 18–25 years old) were obtained during crown-lengthening surgery from the Department of Periodontology at the Ninth People's Hospital, Shanghai Jiao Tong University School of Medicine, between April and September 2015. The Ethical Committee of Shanghai Jiao Tong University approved the protocol for obtaining tissue. Healthy human gingival fibroblasts (hGFs) were isolated and cultured in Dulbecco's modified Eagle's medium (DMEM) (Life Technologies, Carlsbad, CA, USA) containing 100 U/mL penicillin and 100 *μ*g/mL streptomycin (Life Technologies), supplemented with 10% fetal bovine serum (Life Technologies) at 37°C in the presence of 5% CO_2_, and passed every 3-4 days. The lifespan analysis and cumulative population doublings of hGFs were calculated according to a standard culture protocol [18].

### 2.2. Experimental Group Design

#### 2.2.1. Control Group Design

**Figure 1:**
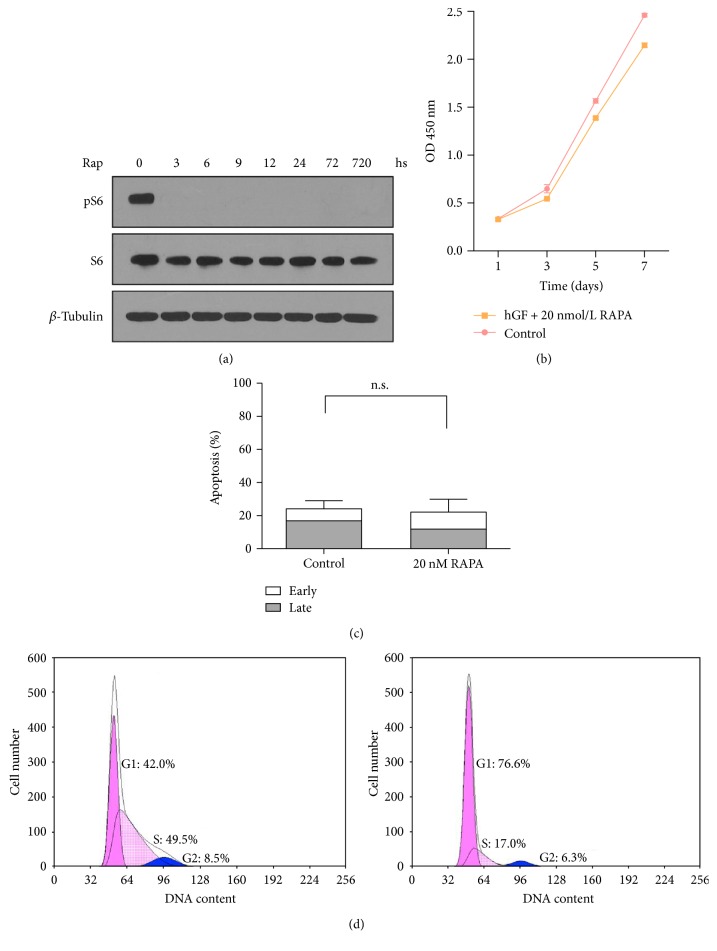
The effect of rapamycin on biological characters. (a) Western blot analysis of hGFs treated with rapamycin for 72 h and 30 d. mTOR inhibition is shown by the levels of pS6. (b) Cell proliferation is detected by Cell Counting Kit-8 (CCK-8) of hGFs for 7 d after rapamycin treatment. (c) Apoptosis of hGFs is examined by an FITC Annexin V Apoptosis Detection Kit (n.s., *P* > 0.05). (d) The cell cycle shows a modest increase in G1 fraction after rapamycin treatment (right).

According to the cumulative population doubling curves of hGFs (shown as the blue line in panel (a) of Figure 1), we defined the low cumulative population doubling levels (CPD ≤ 10) as low-passage hGFs and the high cumulative population doubling levels (CPD > 40) as high-passage hGFs. The high-passage hGFs were continually cultured without any treatment for 30 d for further experiments as the control group.

#### 2.2.2. Experiment Group Design

Short-term mTOR inhibition: high-passage hGFs were treated with 20 nmol/L rapamycin (Sigma-Aldrich Inc., St. Louis, MO, USA) once and harvested after 72 hours for further experiments. This group was defined as *high passage + 3 d RAPA*.

Long-term mTOR inhibition: high-passage hGFs were treated with 20 nmol/L rapamycin for three days with refreshed media and passed when they reached the contact inhibition. hGFs were harvested after 30 day rapamycin treatment for further experiments. This group was defined as *high passage + 30 d RAPA*.

### 2.3. Bacterial Culture and Inoculation

*P. gingivalis* (ATCC 33277) was cultured anaerobically (80% N_2_, 10% H_2_, and 10% CO_2_) in brain-heart infusion (BHI) medium with hemin (5 mg/L). Purity of *P. gingivalis* was checked by Gram staining. hGFs treated with or without rapamycin were challenged with live *P. gingivalis* (MOI = 10) and harvested after 2 hours and 4 hours for further inflammatory experiments.

### 2.4. Cell Proliferation, Cycle, Apoptosis, and Senescence

All of the experiments were performed according to the manufacturer's instructions. Cell proliferation analysis was measured using Cell Counting Kit-8 (CCK-8) (WST-8; Dojindo, Kumamoto, Japan) and absorbance at 450 nm was detected for each well by a microplate reader (BioTek Instruments, Winooski, VT, USA). The effects on cell cycle and cell apoptosis were demonstrated by a Cell Cycle Kit and an FITC-Annexin V Apoptosis Detection Kit (BD Bioscience, San Jose, CA, USA). High-passage hGFs were processed for senescence-associated *β*-galactosidase (SA-*β*-Gal) staining using the SA-*β*-Gal kit (Sigma-Aldrich). The proportion of positive cells was measured by ImageJ software (NIH).

### 2.5. Measurement of Intracellular Reactive Oxygen Species

According to the manufacturer's instruction, ROS was probed with 2′,7′-dichlorodihydrofluorescein diacetate (H_2_DCF-DA; Molecular Probes, Eugene, OR). The intracellular ROS could oxidate DCFH and generate fluorescent product (DCF). We detected intracellular DCF level using a flow cytometer.

### 2.6. Immunofluorescence

The residual proliferative capacity of hGFs was measured by Ki-67 expression through immunofluorescence. Primary antibody (Novocastra-Leica, Wetzlar, Germany) was detected by a specific biotinylated secondary antibody, followed by fluorescein-conjugated avidin (Vector Laboratories, Burlingame, CA, USA). Coverslips were mounted with Fluoroshield with DAPI (Sigma-Aldrich) and *α*-smooth muscle actin (Sigma-Aldrich) to allow visualization of the cell nuclei and cellular morphology.

### 2.7. Western Blotting

After treatment with rapamycin, hGFs were harvested and lysed with RIPA Lysis and Extraction buffer (Pierce Biotechnology, Rockford, IL, USA) and Halt Protease and Phosphatase Inhibitor (Pierce Biotechnology). Protein concentration was quantified using a BCA protein assay kit (Pierce) and fractionated by sodium dodecyl sulphate-polyacryalamide gel electrophoresis (SDS-PAGE), then transferred to polyvinylidene fluoride (PVDF) membranes (Millipore Co., Bedford, MA, USA). The membrane was blocked with 5% nonfat milk and 2% BSA in TBST for 1 h at room temperature and incubated with certain primary antibodies, rabbit anti-human p21 antibody (ab109520; Abcam, Cambridge, UK), rabbit anti-human p16 antibody (ab108349; Abcam), rabbit anti-human phospho-p70 S6 kinase antibody (Thr421/Ser424) (Cell Signaling Technology), rabbit anti-human p70 S6 kinase antibody (Cell Signaling Technology), and rabbit anti-human *β*-tubulin antibody (ab151318; Abcam) at 4°C overnight. The membrane was washed 4 times and incubated with a 1 : 3000 dilution of horseradish peroxidase-conjugated donkey anti-rabbit or anti-mouse IgG antibody (R&D Systems) for 1 h at room temperature. After being washed 3 times with TBST, the immunodetection was developed with the ECL-chemiluminescent kit (Thermo Scientific).

### 2.8. Real-Time PCR

Total RNA was isolated from hGFs using TRIzol Reagent (Life Technologies). cDNA was reverse-transcribed with the TaKaRa Reverse Transcription Kit (TaKaRa, Dalian, China). The cDNAs were then subjected to real-time PCR with the oligonucleotide sequences listed in Table 1. Quantitative PCR was performed on a real-time thermal cycler (Stratagene Mx3000PTM QPCR System, CA, USA) using Power SYBR Green PCR Master Mix (Life Technologies). The relative quantification of each mRNA was calculated after normalization to the human housekeeping gene *GAPDH* as an internal control for quantification using the 2(−Delta Delta C(T)) Method [19].

### 2.9. Enzyme-Linked Immunosorbent Assay

The culture supernatants of each group were collected for determining the concentration of IL-6 and IL-8 using commercially available enzyme-linked immunosorbent assay (ELISA) kits (RayBiotech Inc., Norcross, GA, USA), according to the manufacturer's recommended procedure.

### 2.10. Statistical Analysis

Each assay was performed in triplicate or greater and the means were calculated for analysis. The significant differences in the days before replicative senescence, the maximum cumulative population doublings, and the percentage of apoptotic cells between the control group and rapamycin-treated group were analyzed using unpaired two-tailed Student's *t*-test. The percentage of Ki-67-positive cells, the proportion of SA-*β*-Gal-positive cells, the relative mRNA expression of real-time PCR, and the cytokine levels of ELISA among four groups were statistically analyzed with one-way ANOVA, and a post hoc analysis was performed for the difference in the data between the two groups. The statistical software SPSS (version 22.0, SPSS Inc., IL, USA) was used for statistical analyses. Asterisks indicate the statistical significant levels of NS (*P* > 0.05, ^∗^*P* < 0.05, ^∗∗^*P* < 0.01, and ^∗∗∗^*P* < 0.001). All data are reported as mean ± standard deviation (SD).

## 3. Results

### 3.1. mTOR Inhibition Preserves the Proliferative Capacity of Human Gingival Fibroblasts

**Figure 2:**
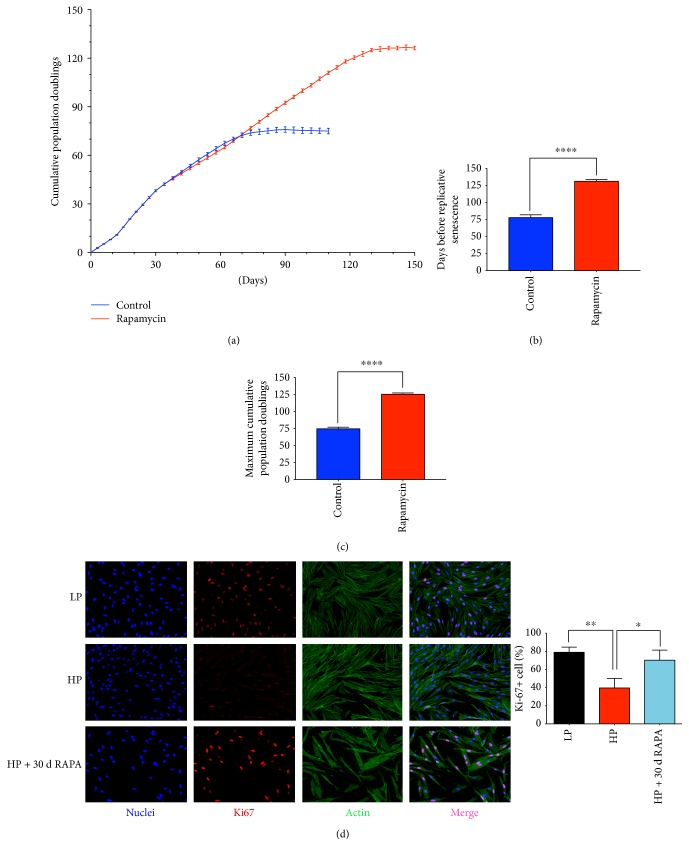
mTOR inhibition protects human gingival fibroblasts from loss of proliferative capacity. (a) Cumulative population doubling from a representative culture of primary hGFs in control conditions or in the continuous presence of rapamycin. Each dot represents a passage. (b, c) Continuous presence of rapamycin doubles the average of the days before replicative senescence and the maximum cumulative population doublings from three different cultures of hGFs. (d) Representative pictures and quantification of the nuclei (blue), the proliferation marker Ki-67 (red), and cytoskeletal protein actin (green). Staining was performed in low passage of hGFs (LP), high passage of hGFs (HP), and high passage of hGFs treated with rapamycin for 30 d (HP + 30 d RAPA). Graphs show the percentage of the Ki-67+ cells (percentage of Ki-67+ cells/total cells) (∗ means *P* < 0.05, ∗∗ means *P* < 0.01, and ∗∗∗∗ means *P* < 0.0001, compared with the high-passage group).

In order to recapitulate the effect of rapamycin on the mTOR signaling, we detected that phospho-p70 S6 kinase was highly phosphorylated in control cells (Figure 2(a)). Notably, mTOR was completely blocked by 20 nmol/L rapamycin for short-term (3 days) and long-term treatments (30 days) (Figure 2(a)). That means 20 nmol/L rapamycin could block the mTOR pathway entirely. Continuous rapamycin treatment dramatically extended the lifespan of hGFs (Figure 1(a)). Specifically, the days before senescence and the total cumulative population doublings were almost doubled compared to those of control cells (Figures 1(b) and 1(c)). The preservation of proliferative potential was paralleled by an increase in Ki-67 expression through immunofluorescence. As shown in Figure 1(d), low-passage fibroblasts displayed a higher proportion of Ki-67 (38% in low passage versus 18% in high passage) staining while the rapamycin-treated group preserved the proliferative potential.

### 3.2. mTOR Inhibition Postpones the Onset of Senescence and Regulates the Expression of Senescence-Associated Markers

**Figure 3:**
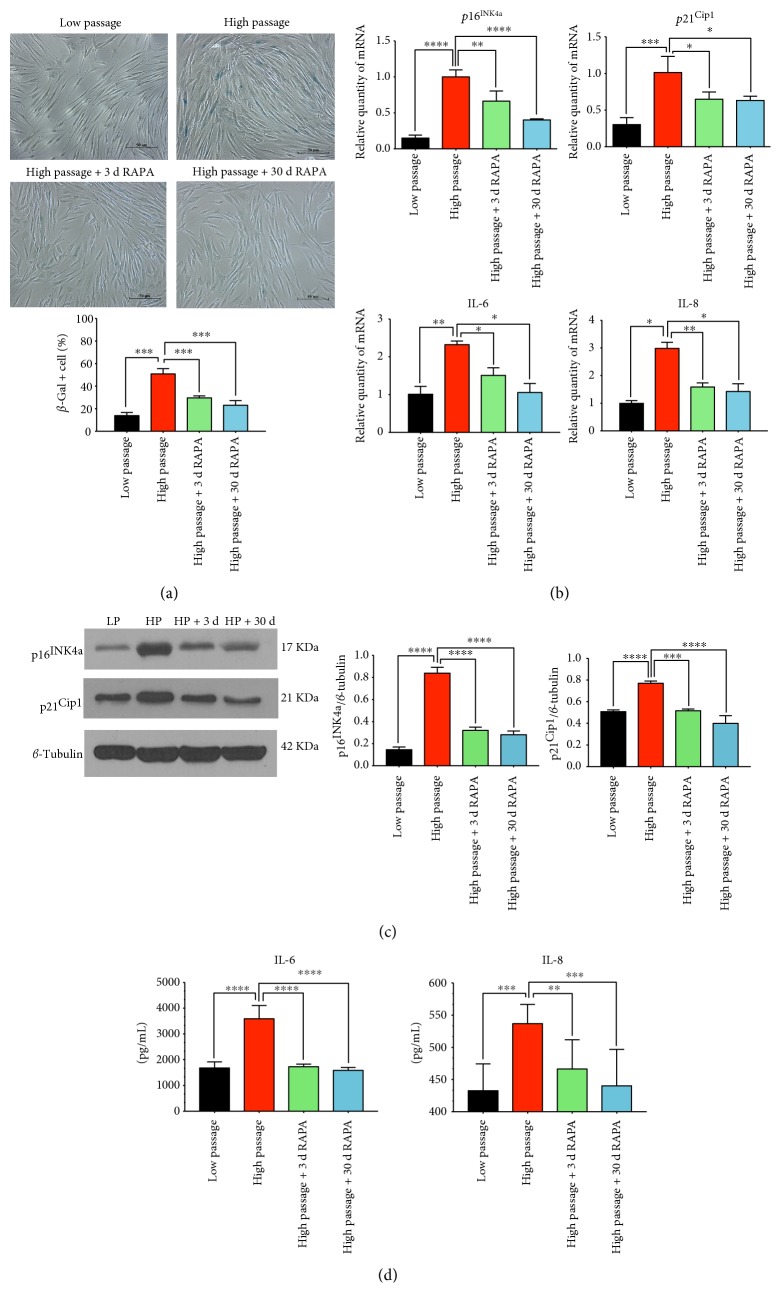
Rapamycin delays the onset of senescence. (a) Proportion with respect to control cells and representative pictures of hGFs positive for senescence-associated *β*-galactosidase (SA-*β*-Gal) 3 days and 30 days after rapamycin treatment. (b) The results of real-time PCR show the mRNA expression of senescence-associated markers p16, p21, IL-6, and IL-8. (c) The result of Western blot shows the protein secretions of senescence-associated markers p16 and p21. (d) The results of ELISA show the cytokine secretions of senescence-associated markers IL-6 and IL-8 (∗ means *P* < 0.05, ∗∗ means *P* < 0.01, ∗∗∗ means *P* < 0.001, and ∗∗∗∗ means *P* < 0.0001, compared with the high-passage group).

hGFs undergo irreversible senescence upon continuous replication [20]. Rapamycin-treated hGFs almost doubled their days before senescence when compared to control cells (Figure 1(b)). The senescence-associated markers, such as the senescence-associated beta-galactosidase (SA-*β*-Gal), the cyclin-dependent kinase (CDK) inhibitors (p16^INK4a^ and p21^CIP1a^), and inflammatory cytokines (IL-6 and IL-8), were detected by SA-*β*-Gal staining, real-time PCR, Western blotting, and ELISA. The SA-*β*-Gal staining was partially decreased in rapamycin-treated groups (both short-term and long-term treatments; Figure 3(a)), but the flat cell morphology did not change (Figure 1(d), shown by actin staining). The results from real-time PCR (Figure 3(b)) showed that mTOR inhibition could significantly reduce the mRNA expression of *p16^INK4a^*, *p21^CIP1a^*, *IL-6*, and *IL-8* compared with the untreated high-passage hGF group. The results from Western blotting (Figure 3(c)) and ELISA (Figure 3(d)) were consistent with real-time PCR. Analysis of these results indicates that rapamycin partially reversed the senescent progress in hGFs.

### 3.3. mTOR Inhibition Alleviates Oxidative Stress in Human Gingival Fibroblasts

**Figure 4:**
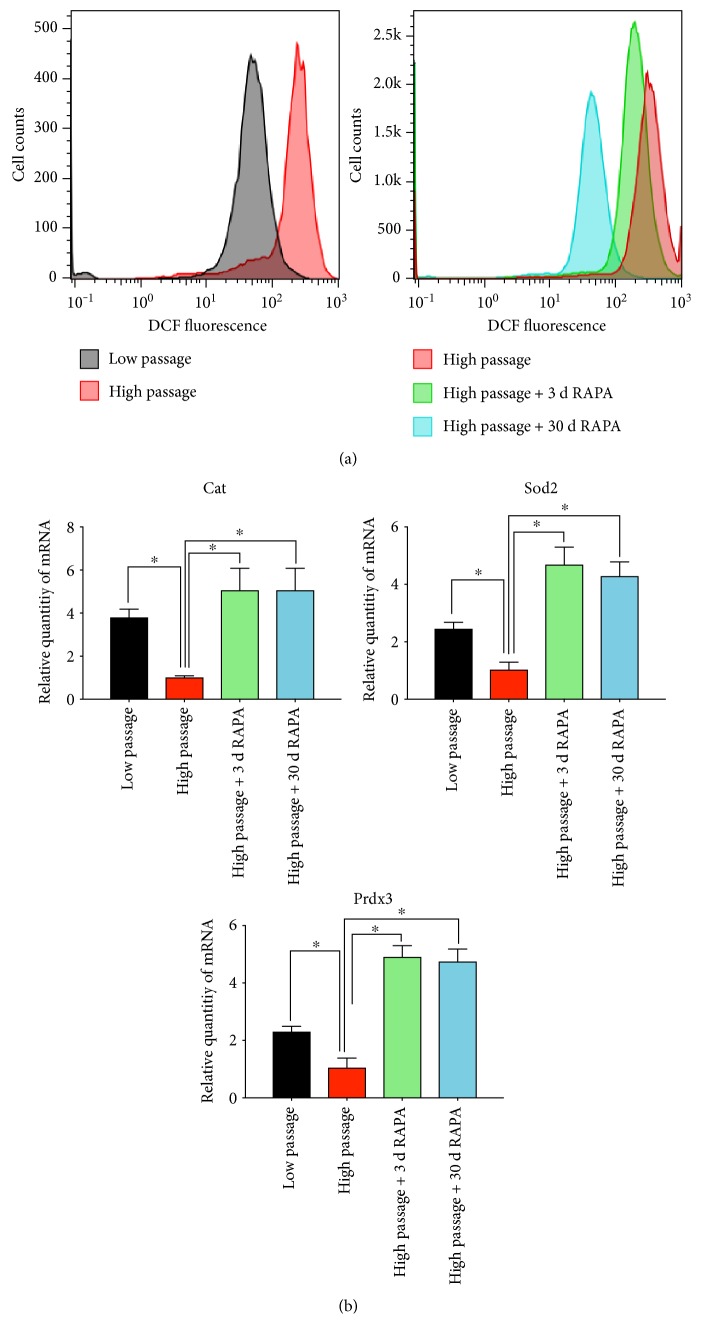
mTOR inhibition alleviates oxidative stress in human gingival fibroblasts. (a) FACS analysis of reactive oxygen species (ROS) levels in low-passage hGFs and high-passage hGFs pretreated or not (control) with rapamycin after 3 or 30 days. (b) The results of real-time PCR show the mRNA expression of antioxidant components Cat, Sod2, and Prdx3 (∗ means *P* < 0.05, compared with the high-passage group).

DCF fluorescent level showed the content of intracellular reactive oxygen species (ROS), and the DCF fluorescent level of the cells was gradually increased as the continuous culture (Figure 4(a), left panel). While treated with rapamycin, intracellular ROS level of high-passage hGFs decreased (Figure 4(a), right panel). And long-term treatment may decrease the ROS level even more compared with short-term treatment (Figure 4(a), right panel). The results of real time-PCR (Figure 4(b)) show that the mRNA expression of the antioxidant components catalase (*Cat*), manganese superoxide dismutase (*Sod2*), and peroxiredoxin-3 (*Prdx3*) decrease while in continuously replicative culture (high passage versus low passage). Treated with rapamycin, however, high-passage hGFs expressed the antioxidant components more in order to eliminate the intracellular ROS.

### 3.4. Rapamycin Enhances the Anti-Inflammatory Ability of Human Gingival Fibroblasts

**Figure 5:**
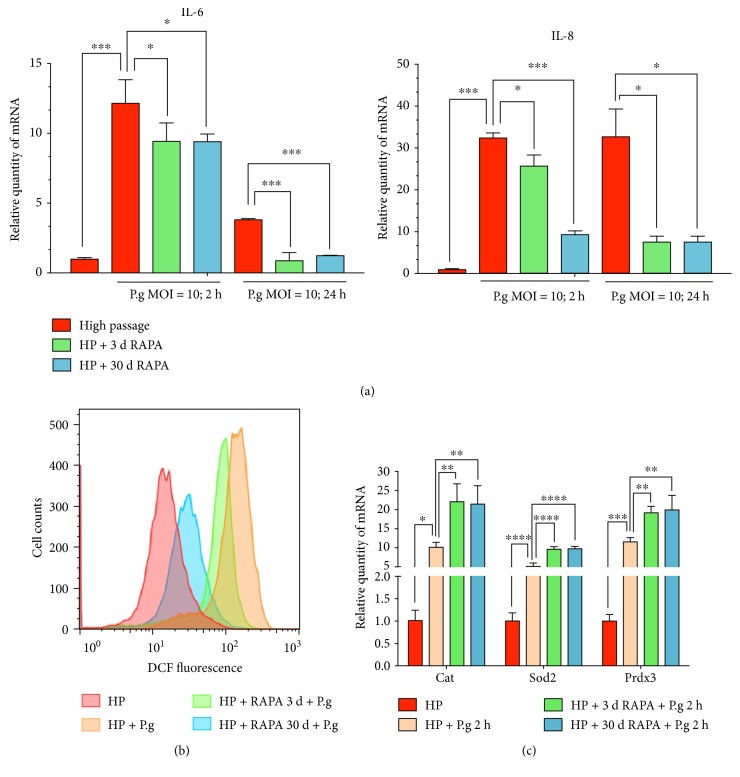
Rapamycin enhances the anti-inflammatory ability of human gingival fibroblasts. (a) The results of real time-PCR show the mRNA expression of inflammatory cytokines IL-6 and IL-8. Both short- and long-term treatments of rapamycin can ease the inflammatory response caused by *P. gingivalis* infection. (b) FACS analysis shows the reactive oxygen species (ROS) levels in hGFs. Rapamycin treatment alleviates the intracellular ROS level in hGFs caused by *P. gingivalis*. (c) The results of real-time PCR show (right panel) the mRNA expression of antioxidant components Cat, Sod2, and Prdx3 (∗ means *P* < 0.05, ∗∗ means *P* < 0.01, ∗∗∗ means *P* < 0.001, and ∗∗∗∗ means *P* < 0.0001, compared with the control group).

We infected high-passage hGFs with *P. gingivalis* (ATCC 33277) (MOI = 100, 2 hours and 24 hours). The results of real time-PCR (Figure 5(a)) showed that mRNA expression of the inflammatory cytokines IL-6 and IL-8 increased while infected with *P. gingivalis*. Both long-term and short-term rapamycin treatments may have less expression of *IL-6* and *IL-8* (2 hours). And rapamycin treatment may help to alleviate the inflammatory response (24 hours). After infected with *P. gingivalis* (MOI = 100, 2 hours), the intracellular ROS of hGFs were detected by FACS.  Figure 5(b) shows that the ROS level of hGFs was increased while infected by *P. gingivalis* and pretreatment with rapamycin for 3 days may eliminate the intracellular ROS. The results of real time-PCR (Figure 5(c)) showed that hGFs might express antioxidant components when attacked by *P. gingivalis*. Pretreatment with rapamycin for 3 days enhanced the expression of antioxidant components.

## 4. Discussion

Using replicative senescent human gingival fibroblasts, we demonstrated that the mTOR inhibition partially reversed the aging process and elevated the anti-inflammatory ability of gingival fibroblasts. As shown in results, inhibition of mTOR activity with the selective inhibitor, rapamycin, preserved the proliferative capacity, delayed the onset of senescence, and remitted the inflammatory response through alleviating oxidative stress. These results are consistent with the findings that the mTOR signaling plays a crucial role in cell growth and cellular metabolism [13].

The pivotal aspect of cellular senescence is a proliferation arrest but metabolic activity maintained, resulting in decreasing tissue regenerative capacity [21, 22]. Consecutive cell divisions may accompany with telomere shortening leading to replicative senescence [23]. Figuratively speaking, our results suggested that mTOR inhibition preserved the Ki-67 staining (a well-known marker of proliferation; Figure 1(d)) but the well-defined flat cell morphology did not change (Figure 2(d), shown by actin staining) in replicative senescent hGFs. As the lifespan curve (Figures 2(a), 2(b), and 2(c)) shows, a long-term (30 d) treatment approximately doubled the days before replicative senescence and the maximum cumulative population doublings. Senescent cells that accumulate during aging may have profound meanings at organism level, and the clearance of accumulated senescent cells may help to prevent tissue disorder and extend health span [24]. It is an appealing hypothesis that rapamycin treatment may contribute to the long-lived phenotype of mice by reducing the accumulation of senescent cells [25] or by reducing the upstream of mTOR signaling pathway activity [26]. But the mechanism between mTOR signaling and telomere shortening still remains to be explored.

Senescent cells are hypothesized to involve disruption of tissue homeostasis because of a multifarious senescence-associated secretory phenotype (SASP). The secretion of various typical cytokines, chemokines, and growth factors that can disrupt tissue microenvironments and function accrued when cell reached replicative senescence, accompanied with an irreversible proliferation arrest [21]. SASP mediates the diverse effects of senescence on the tissue microenvironment. Our findings that inhibition of mTOR in replicative senescent hGFs partially decreased the SA-*β*-Gal staining (Figure 3(a)) and reduced the cytokine (IL-6 and IL-8; Figures 3(b) and 3(d)) expression appear consistent with other reports that rapamycin reduces SASP in different kinds of cellular senescence [27, 28]. The mechanism of mTOR controlling the SASP seems to modulate gene transcription and mRNA translation and stabilization [21, 27, 29].

mTOR are composed of two biochemically and functionally distinct complexes, mTOR complex 1 (mTORC1) and mTOR complex 2 (mTORC2), existing in all mammalian cells. The two are characterized by differential sensitivity to rapamycin. mTORC1 could be inhibited directly and acutely by rapamycin, while mTORC2 could only be affected by a chronic administration of rapamycin [30]. It is said that mTORC1 controls protein synthesis and responses to multifarious cellular stresses, while mTORC2 regulates cell survival. Our results showed that short-term (3 d) rapamycin treatment could block the cell cycle (Figure 2(d)) and change the senescent-associated mRNA translation and protein expression (p16^INK4a^, p21^CIP1a^, IL-6, and IL-8; Figure 3). It indicated that a short-term rapamycin administration might implicate mTORC1 and manipulate senescence positively. Numerous studies revealed that the results of handling mTOR activity during senescence seemed to vary resting with the model [28, 31, 32]. We hypothesize the effects of short-term rapamycin treatment may occur by inhibiting mTORC1, while a long-term rapamycin treatment may involve the functions of mTORC2.

CDK inhibitors are induced by myriad cellular stresses. p21^CIP1a^ and p16^INK4a^ are key regulators of senescence causing an irreversible arrest of the cell cycle [23]. Cells go to senesce and gradually lose proliferative capacity while cell cycle is irreversibly arrested but metabolic activity is not. Both in human and in rodent cell lines, studies show that rapamycin spectacularly released the stress induced by ectopic p21^CIP1a^ and p16^INK4a^ [31], which certified our findings that mTOR inhibition resulted in downregulation of the expression of p21^CIP1a^ and p16^INK4a^ in replicative senescent hGFs, and lower levels of p21^CIP1a^ and p16^INK4a^ are sustained over long-term treatment. Rapamycin treatment may slightly delay cell cycle (Figure 2(d)) that led to preserving the proliferative potential.

It has been reported that reducing mTOR activity might increase lifespan in human fibroblasts by enhancing mitochondrial membrane potential [11, 33, 34]. Intracellular ROS is produced mainly in the mitochondria. Mitochondrial integrity interacts with senescence. Impaired mitochondrial function is crucial in age-related oxidative damage and a potential driver of aging phenotypes in vitro and generates telomere attrition in vivo [22]. Telomerase activity has a consequent impact on cellular function and potentially influences mitochondrial function through a p53-regulated pathway [35]. We demonstrated that rapamycin partially remitted oxidative stress which occurred in senescence. Replicative senescent hGFs had a higher level of intracellular ROS compared with the control (Figure 4(a), left panel). Both short- and long-term rapamycin treatments could improve the ability of eliminating intracellular ROS formation and oxidative stress (Figure 4(a), right panel) through increasing genes encoding mitochondrial antioxidant component expression (Figure 4(b)).

The gingiva, the frontline of oral natural barriers, may be more susceptible to periodontal pathogens while aging. Study showed that rapamycin prevented aged mice from pneumonia through lowering the bacterial ligand expression [36]. So we also examined the anti-inflammatory ability of hGFs against *P. gingivalis* when pretreated with rapamycin. It is an intriguing hypothesis that when human gingival epithelial cells were infected with live *P. gingivalis*, intracellular ROS level rapidly increased [37], similar to our in vitro results in human gingival fibroblasts (Figure 5(a)). A partial rejuvenation of the aging gingiva may have important implications at antimicrobial resistance, given the rapamycin pretreatment ameliorated the oxidative stress caused by live *P. gingivalis* (Figure 5(b)). mTOR inhibition may preserve mitochondrial function, which in turn remitted the inflammatory reaction. The mechanism of anti-inflammation of mTOR inhibition remains to be further studied.

mTOR signaling is triggered by various environmental stimuli and modulates several known longevity factors in a complex signaling network including an insulin/IGF-1-like axis [12]. Insulin/IGF-1 is recognized at the cell surface by an IGF receptor and provides the primary extracellular regulation of longevity and cellular proliferation. IGF receptor loss has been shown to increase lifespan in mice and worms [38]. A study showed that an intact insulin/IGF-1 axis was essential to maintain health span and overexpressed IGF-1 associated with increased pathology [39]. Taken together, lifespan regulation via the mTOR signaling pathway is conserved, growth hormone and IGF-1 should be clinically applied with caution.

It has been proved that mTOR inhibition could postpone the aging process in a rodent model. There are still few studies showing the effects of mTOR inhibition on a primate model. The present study provides an innovative approach that may help to preserve proliferative potential and improve the anti-inflammatory ability of human aging gingival tissues through releasing the intracellular oxidative stress. Future studies may focus on the target gene involved in mTOR inhibition to ameliorate the aging process and the systemic effect of TOR signaling in multicellular organisms.

## Figures and Tables

**Table 1:** Primer sequence used for polymerase chain reaction amplifications.

Gene	Primer	Sequences (5′ to 3′)
p16INK4a	Forward	CTCCGGAAGCTGTCGACTTC
	Reverse	TTCTGCCATTTGCTAGCAGTGT
P21Cip1	Forward	CGATGGAACTTCGACTTTGTCA
	Reverse	GCACAAGGGTACAAGACAGTG
Cat	Forward	TGTTGCTGGAGAATCGGGTTC
	Reverse	TCCCAGTTACCATCTTCTGTGTA
Sod2	Forward	GCTCCGGTTTTGGGGTATCTG
	Reverse	GCGTTGATGTGAGGTTCCAG
Prdx3	Forward	ACTGTGAAGTTGTCGCAGTCT
	Reverse	CACACCGTAGTCTCGGGAAA
IL-6	Forward	ACTCACCTCTTCAGAACGAATTG
	Reverse	CCATCTTTGGAAGGTTCAGGTTG
IL-8	Forward	ACTGAGAGTGATTGAGAGTGGAC
	Reverse	AACCCTCTGCACCCAGTTTTC
GAPDH	Forward	CCACTCCTCCACCTTTGAC
	Reverse	ACCCTGTTGCTGTAGCCA
